# Benzene-1,3,5-triyl tribenzoate

**DOI:** 10.1107/S1600536813031462

**Published:** 2013-11-27

**Authors:** Peter W. R. Corfield, Amy M. Balija

**Affiliations:** a Department of Chemistry, Fordham University, 441 East Fordham Road, Bronx, NY 10458, USA

## Abstract

The title compound, C_27_H_18_O_6_, commonly known as phloroglucinol tribenzoate, is a standard unit for the family of benzyl ether dendrimers. The central phloroglucinol residue is close to planar, with out-of-plane distances for the three oxygen atoms of up to 0.095 (3) Å, while the three attached benzoate groups are approximately planar. One benzoate group is twisted [C—C—O—C torsion angle = 98.2 (3)°] from the central plane, with its carbonyl O atom 2.226 (4) Å above that plane, while the other two benzoate groups are twisted in the opposite direction [C—C—O—C torsion angles = 24.7 (2) and 54.8 (2)°], so that their carbonyl O atoms are on the other side of, and closer to the central plane, with distances from the plane of 1.743 (4) and 1.206 (4) Å. One benzoate group is disordered between two conformers, with occupancies of 86.9 (3) and 13.1 (3)%, related by a 143 (1)° rotation about the bond to the central benzene ring. The phenyl groups of the two conformers occupy the same space. The mol­ecule packs in the crystal with two of the three benzoate phenyl rings stacked parallel to symmetry-related counterparts, with perpendicular distances of 3.715 (5) and 3.791 (5) Å. The parallel rings are slipped away from each other, however, with centroid–centroid distances of 4.122 (2) and 4.363 (2) Å, respectively.

## Related literature

For a review of structural features of specific dendrimers, see: Stadler (2010 ▶). For related crystal structures, see: Pigge *et al.* (2010 ▶); Shi & Zhang (2006 ▶); Sasvari & Parkanyi (1980 ▶). For related papers on the properties and synthesis of dendrimers, see: Monaco *et al.* (2013 ▶); Moore & Stupp (1990 ▶); Nagvekar & Gibson (1997 ▶).
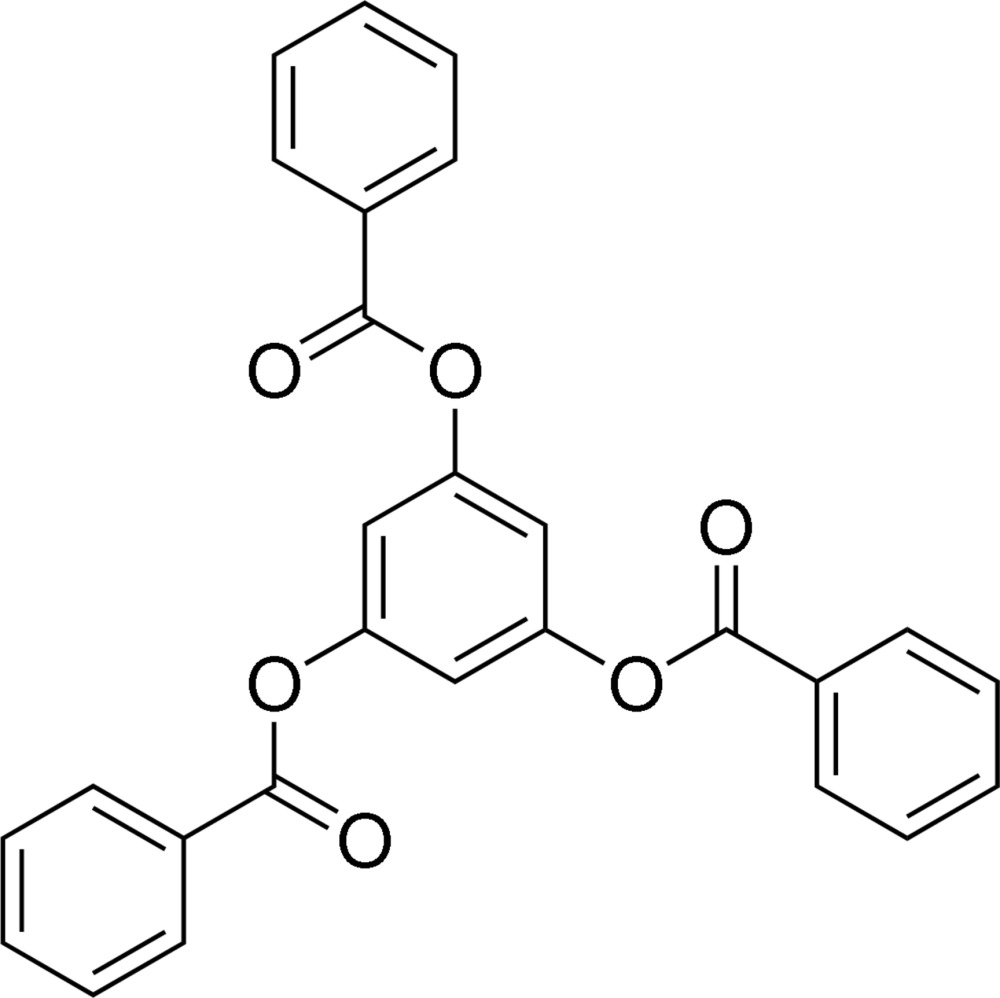


## Experimental

### 

#### Crystal data

C_27_H_18_O_6_
*M* *_r_* = 438.41
Monoclinic, 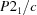

*a* = 23.128 (5) Å
*b* = 6.332 (2) Å
*c* = 15.030 (3) Å
β = 103.22 (2)°
*V* = 2142.8 (9) Å^3^
*Z* = 4
Mo *K*α radiation
μ = 0.10 mm^−1^
*T* = 295 K
0.4 × 0.4 × 0.13 mm

#### Data collection

Enraf–Nonius CAD-4 diffractometer
4950 measured reflections
3775 independent reflections
2096 reflections with *I* > 2σ(*I*)
*R*_int_ = 0.021
3 standard reflections every 120 min intensity decay: 0.5 (4)%

#### Refinement

*R*[*F* ^2^ > 2σ(*F* ^2^)] = 0.040
*wR*(*F* ^2^) = 0.114
*S* = 1.03
3775 reflections
295 parameters
2 restraints
H-atom parameters constrained
Δρ_max_ = 0.15 e Å^−3^
Δρ_min_ = −0.16 e Å^−3^

### 

Data collection: *CAD-4 Software* (Enraf–Nonius, 1989 ▶); cell refinement: *CAD-4 Software*; data reduction followed procedures in Corfield *et al.* (1973 ▶) and data were averaged with a local version of *SORTAV* (Blessing, 1989 ▶); program(s) used to solve structure: *SHELXS97* (Sheldrick, 2008 ▶); program(s) used to refine structure: *SHELXL97* (Sheldrick, 2008 ▶); molecular graphics: *ORTEPIII* (Burnett & Johnson, 1996 ▶); software used to prepare material for publication: *SHELXL97*.

## Supplementary Material

Crystal structure: contains datablock(s) I. DOI: 10.1107/S1600536813031462/pk2503sup1.cif

Structure factors: contains datablock(s) I. DOI: 10.1107/S1600536813031462/pk2503Isup2.hkl

Supplementary material file. DOI: 10.1107/S1600536813031462/pk2503Isup3.cml

Additional supplementary materials:  crystallographic information; 3D view; checkCIF report

## Figures and Tables

**Fig. 1.**
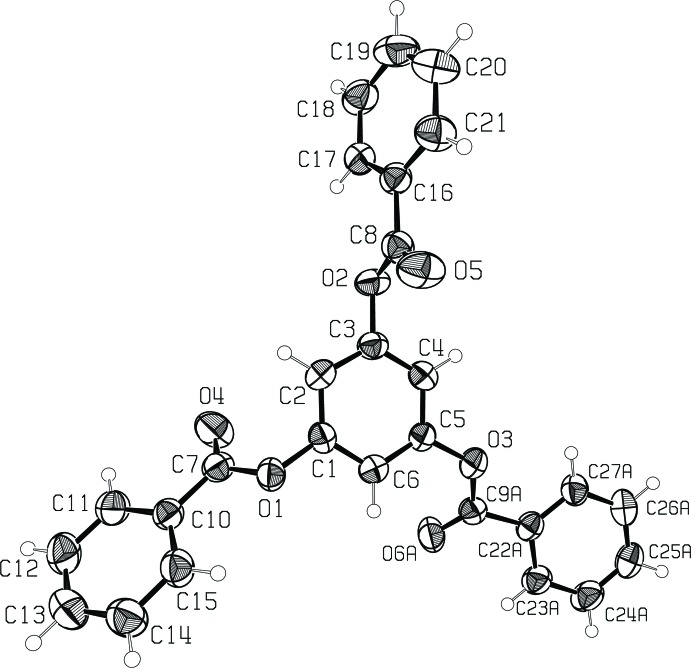
The ORTEPIII drawing of the phloroglucinol tribenzoate molecule, with ellipsoids at the 50% level. The view is perpendicular to the central phenyl group, atoms C1–C6. Only the major component of the disordered benzoate group is shown, with atoms labeled A.

**Fig. 2.**
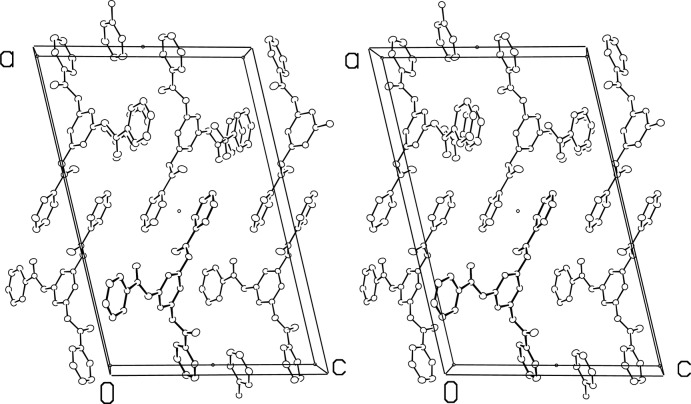
Packing diagram for the phloroglucinol tribenzoate structure, showing how two of the benzoate phenyl groups stack around centers of symmetry at (0,0,1/2) and (1/2,1/2,1/2). The molecule at (x,y,z) is boldly outlined. Ellipsoids are at the 30% level.

**Fig. 3.**
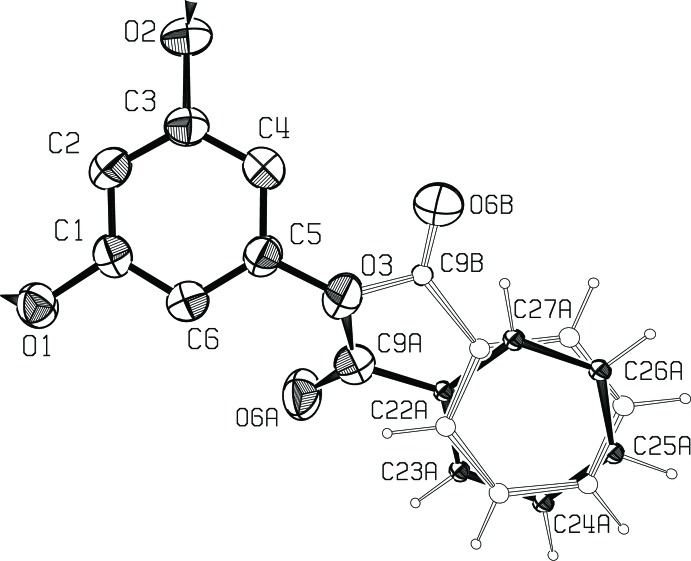
Part of the molecule of phloroglucinol tribenzoate, showing the disordered benzoate group.

## supplementary crystallographic information

### 1. Comment

Dendrimers are macromolecules whose composition and molecular weights are well defined. The present work is part of a systematic study examining how functional groups within a dendrimer family influence the ability to remove small organic pollutants from an aqueous environment (Monaco *et al.* 2013). The title compound is a model for larger dendrimer systems.

The central phloroglucinol residue is close to planar, with out-of-plane distances for the three oxygen atoms varying up to 0.09 Å. Deviations from planarity for the three benzoate groups are larger, with the carbonyl groups twisting to make their oxygen atoms 0.3–0.4 Å from the planes of the phenyl groups (see Fig.1). The benzoate group (C16–C21,C8,O5) is twisted approximately perpendicular to the central plane, with torsional angle C2–C3–O2–C8 = 98.2 (3)°, forcing its carbonyl oxygen atom 2.226 (4) Å Above the central plane. The other two benzoate groups are twisted less, and in the opposite sense, with torsional angles C6–C1–O1–C7 = -125.2 (2) and C4–C5–O3–C9A = -146.3 (2)°, and carbonyl oxygen atoms on the other side of and closer to the central plane. Similar torsional angles were observed in one of the two similar molecules in Shi and Zhang (2006), and in Sasvari and Parkanyi (1980).

Fig. 2 shows the packing of the phloroglucinol tribenzoate molecules in the crystal. There may be some interactions between aromatic rings C10–C15 and C16–C21 and their parallel counterparts related by centers of symmetry at 1/2,1/2,1/2 and 0,0,1/2. The perpendicular distances between the planes of the symmetry related rings are 3.715 (5) Å and 3.791 (5) Å respectively. There are few close contacts between the parallel rings, however, as the rings are slipped from direct opposition by 1.78 and 2.15 Å respectively. Indeed the shortest intermolecular contacts in the crystal occur between hydrogen atoms on parallel rings displaced by one unit cell in the direction of the b axis: H21.. H21 (-*x*,1 - *y*,1 - *z*) = 2.32 Å and H11.. H11(1 - *x*,-*y*,1 - *z*) = 2.74 Å. The disordered benzoate does not show any interaction between parallel phenyl rings.

### 2. Experimental

The synthesis described below was performed under an argon gas atmosphere with oven dried glassware. Reagents were obtained from Aldrich. The reagent 2-(dimethylamino)pyridinium *p*-toluenesulfonate (DPTS) was synthesized as reported previously (Moore *et al.*, 1990). Solvents and reagents were used without further purification except for the following: dichloromethane was distilled from CaH_2_ and phloroglucinol dihydrate was azeotroped 5 times with toluene prior to use. Eluent solvent ratios are reported in *v*/*v*.

^1^H NMR spectra were recorded at 300 MHz and ^13^C NMR spectra were recorded at 75 MHz on a Bruker AV-300 High Performance Digital NMR Spectrometer. Chemical shifts are reported in parts per million (p.p.m.) and coupling constants are reported in Hertz (Hz). ^1^H NMR spectra obtained in CDCl_3_ were referenced to 7.26 p.p.m. and ^13^C NMR spectra obtained in CDCl_3_ were referenced to 77.2 p.p.m.. Mass spectra were obtained from the University of Illinois Mass Spectrometry Center (Micromass Q-Tof Ultra, ESI).

The preparation of phloroglucinol tribenzoate was performed as follows: To a solution of 0.25 g (2.05 mmol) of benzoic acid in 10 ml of dichloromethane was added 0.08 g (0.62 mmol) of phloroglucinol, 0.47 g (2.26 mmol) of *N*,*N*'-dicyclohexylcarbodiimide, and 0.63 g (2.26 mmol) of DPTS. The reaction was stirred overnight, filtered, and washed with cold dichloromethane. After the solvent was removed *in vacuo*, the resulting material was purified by silica gel column chromatography (gradient system 2:1 petroleum ether:dichloromethane → 1:1 petroleum ether:dichloromethane) to obtain 0.16 g (58% yield) of the product as a white solid. ^1^H NMR (CDCl_3_): σ 8.22 (d, *J* = 7.1, 6H), 7.67 (tt, *J* = 7.4, 1.6, 3H), 7.54 (t, *J* = 7.6, 6H), 7.19 (s, 3H). ^13^C NMR (CDCl_3_): σ 164.6, 151.8, 134.1, 130.4, 129.2, 128.9, 113.5. MS-ESI: *m/z* [*M* + Na+K]^+2^: 500.3. Spectral data were similar to previously reported data (Nagvekar *et al.*, 1997). Single crystals appeared upon slow evaporation of a solution of phloroglucinol tribenzoate in dichloromethane.

### 3. Refinement

Refinements with anisotropic temperature factors for C and O atoms and constrained hydrogen atom parameters converged smoothly to *R*(F^2^>2σ)=0.0563 for 299 variables.

The difference Fourier synthesis at this point showed two peaks of 0.78 and 0.55 e Å^3^ in the vicinity of the ester oxygen O3, with no other peak above 0.21 e Å^3^. We have interpreted these two peaks with a partially disordered structure in which the benzoate group O3–C9A(–O6)-(C22–C27) is rotated 149 (1)° about C5–O3 so that C9B and O6B, alternatives to C9A and O6A, fit on the two peaks. The rotated ring C22B–C27B occupies the same space as the original C22A–C27A ring. The relationship of the disordered rings is illustrated in Fig. 3.

In final refinements modeling this disorder, the bond lengths for O3—C9B and C9B—C27B were constrained, to avoid their refinement to unreasonable values. The disordered phenyl group C22B—C27B was constrained to a rigid hexagon with bond lengths 1.378 Å, a value chosen from results of refinements where this distance was increased incrementally. Joint anisotropic temperature factors were assigned for corresponding carbon atoms in the disordered phenyl groups. Atom O6B was allowed to vibrate anisotropically. To reduce the number of parameters varied, the benzoate phenyl groups C10—C15 and C16—C21 were also constrained as rigid hexagons. With these constraints, refinement converged with *R*(F^2^>2σ)=0.0401 for 295 variables. Occupancy factors for the disordered groups are 86.9 (3)% and 13.1 (3)%. The new final difference Fourier synthesis showed no peaks above 0.16 e Å^3^. Use of further restraints on distances in the disordered benzoate group did not improve the geometry.

### Crystal data

**Table:**

C_27_H_18_O_6_	*F*(000) = 912
*M**_r_* = 438.41	*D*_x_ = 1.359 Mg m^−^^3^
Monoclinic, *P*2_1_/*c*	Mo *K*α radiation, λ = 0.71070 Å
Hall symbol: -P 2ybc	Cell parameters from 25 reflections
*a* = 23.128 (5) Å	θ = 3.6–20.3°
*b* = 6.332 (2) Å	µ = 0.10 mm^−^^1^
*c* = 15.030 (3) Å	*T* = 295 K
β = 103.22 (2)°	Plate cut from large crystal, colourless
*V* = 2142.8 (9) Å^3^	0.4 × 0.4 × 0.13 mm
*Z* = 4	

### Data collection

**Table:**

Enraf–Nonius CAD-4 diffractometer	*R*_int_ = 0.021
Radiation source: fine-focus sealed tube	θ_max_ = 25.0°, θ_min_ = 1.8°
Graphite monochromator	*h* = −27→26
θ/2θ scans	*k* = 0→7
4950 measured reflections	*l* = 0→17
3775 independent reflections	3 standard reflections every 120 min
2096 reflections with *I* > 2σ(*I*)	intensity decay: 0.5(4)

### Refinement

**Table:**

Refinement on *F*^2^	Hydrogen site location: inferred from neighbouring sites
Least-squares matrix: full	H-atom parameters constrained
*R*[*F*^2^ > 2σ(*F*^2^)] = 0.040	*w* = 1/[σ^2^(*F*_o_^2^) + (0.*P*)^2^ + 0.450*P*] where *P* = (*F*_o_^2^ + 2*F*_c_^2^)/3
*wR*(*F*^2^) = 0.114	(Δ/σ)_max_ < 0.001
*S* = 1.03	Δρ_max_ = 0.15 e Å^−^^3^
3775 reflections	Δρ_min_ = −0.16 e Å^−^^3^
295 parameters	Extinction correction: *SHELXL97* (Sheldrick, 2008), Fc^*^=kFc[1+0.001xFc^2^λ^3^/sin(2θ)]^-1/4^
2 restraints	Extinction coefficient: 0.0067 (7)
Primary atom site location: structure-invariant direct methods	

### Special details

**Table:**

Geometry. All e.s.d.'s (except the e.s.d. in the dihedral angle between two l.s. planes) are estimated using the full covariance matrix. The cell e.s.d.'s are taken into account individually in the estimation of e.s.d.'s in distances, angles and torsion angles; correlations between e.s.d.'s in cell parameters are only used when they are defined by crystal symmetry. An approximate (isotropic) treatment of cell e.s.d.'s is used for estimating e.s.d.'s involving l.s. planes.
Refinement. Refinement of *F*^2^ against ALL reflections. The weighted *R*-factor *wR* and goodness of fit *S* are based on *F*^2^, conventional *R*-factors *R* are based on *F*, with *F* set to zero for negative *F*^2^. The threshold expression of *F*^2^ > σ(*F*^2^) is used only for calculating *R*-factors(gt) *etc*. and is not relevant to the choice of reflections for refinement. *R*-factors based on *F*^2^ are statistically about twice as large as those based on *F*, and *R*- factors based on ALL data will be even larger.

### Fractional atomic coordinates and isotropic or equivalent isotropic displacement parameters (Å^2^)

**Table:**

	*x*	*y*	*z*	*U*_iso_*/*U*_eq_	Occ. (<1)
O1	0.35661 (7)	0.5340 (3)	0.49441 (10)	0.0470 (4)	
O2	0.15556 (7)	0.3414 (3)	0.35077 (11)	0.0486 (4)	
O3	0.24069 (7)	0.9621 (3)	0.25815 (12)	0.0538 (5)	
O4	0.37050 (8)	0.2080 (3)	0.44526 (14)	0.0740 (6)	
O5	0.11874 (8)	0.5541 (3)	0.44322 (14)	0.0719 (6)	
C1	0.30356 (10)	0.5610 (4)	0.42800 (15)	0.0405 (6)	
C2	0.25623 (10)	0.4259 (4)	0.42122 (16)	0.0438 (6)	
H2	0.2590	0.3050	0.4571	0.053*	
C3	0.20481 (10)	0.4767 (4)	0.35957 (16)	0.0421 (6)	
C4	0.19981 (10)	0.6514 (4)	0.30364 (16)	0.0436 (6)	
H4	0.1646	0.6809	0.2614	0.052*	
C5	0.24860 (10)	0.7809 (4)	0.31228 (15)	0.0416 (6)	
C6	0.30092 (10)	0.7405 (4)	0.37541 (15)	0.0429 (6)	
H6	0.3333	0.8312	0.3822	0.051*	
C7	0.38779 (10)	0.3519 (4)	0.49572 (17)	0.0455 (6)	
C8	0.11408 (10)	0.3985 (4)	0.39679 (17)	0.0466 (6)	
C10	0.44494 (5)	0.3584 (3)	0.56490 (10)	0.0428 (6)	
C11	0.47896 (7)	0.1775 (2)	0.57685 (11)	0.0577 (7)	
H11	0.4646	0.0550	0.5453	0.069*	
C12	0.53426 (7)	0.1781 (3)	0.63561 (13)	0.0669 (8)	
H12	0.5572	0.0560	0.6437	0.080*	
C13	0.55554 (6)	0.3595 (3)	0.68242 (11)	0.0650 (8)	
H13	0.5929	0.3599	0.7221	0.078*	
C14	0.52152 (7)	0.5404 (3)	0.67047 (11)	0.0642 (8)	
H14	0.5359	0.6629	0.7021	0.077*	
C15	0.46622 (7)	0.5399 (2)	0.61171 (12)	0.0551 (7)	
H15	0.4433	0.6620	0.6036	0.066*	
C16	0.06392 (6)	0.2479 (2)	0.38278 (11)	0.0440 (6)	
C17	0.06666 (6)	0.0496 (3)	0.34617 (11)	0.0513 (7)	
H17	0.1011	0.0056	0.3296	0.062*	
C18	0.01834 (8)	−0.0835 (2)	0.33404 (12)	0.0607 (8)	
H18	0.0202	−0.2173	0.3093	0.073*	
C19	−0.03272 (7)	−0.0183 (3)	0.35852 (13)	0.0720 (9)	
H19	−0.0653	−0.1081	0.3503	0.086*	
C20	−0.03546 (6)	0.1800 (3)	0.39513 (13)	0.0749 (9)	
H20	−0.0699	0.2239	0.4116	0.090*	
C21	0.01286 (7)	0.3131 (2)	0.40725 (12)	0.0599 (8)	
H21	0.0110	0.4469	0.4320	0.072*	
C22A	0.26207 (19)	1.2348 (5)	0.1664 (2)	0.0428 (10)	0.869 (3)
C23A	0.30488 (14)	1.3733 (8)	0.1497 (2)	0.0522 (9)	0.869 (3)
H23A	0.3449	1.3461	0.1738	0.063*	0.869 (3)
C24A	0.2879 (2)	1.5522 (7)	0.0970 (3)	0.0608 (9)	0.869 (3)
H24A	0.3165	1.6471	0.0872	0.073*	0.869 (3)
C25A	0.2294 (3)	1.5889 (6)	0.0596 (3)	0.0573 (9)	0.869 (3)
H25A	0.2183	1.7081	0.0236	0.069*	0.869 (3)
C26A	0.18673 (18)	1.4519 (10)	0.0746 (3)	0.0561 (9)	0.869 (3)
H26A	0.1470	1.4774	0.0478	0.067*	0.869 (3)
C27A	0.20245 (17)	1.2766 (7)	0.1291 (3)	0.0487 (10)	0.869 (3)
H27A	0.1733	1.1866	0.1408	0.058*	0.869 (3)
C9A	0.28368 (12)	1.0471 (5)	0.22254 (18)	0.0428 (7)	0.869 (3)
O6A	0.33266 (8)	0.9761 (4)	0.23489 (14)	0.0620 (7)	0.869 (3)
C27B	0.2213 (18)	1.234 (3)	0.1497 (18)	0.0487 (10)	0.131 (3)
C22B	0.2801 (15)	1.294 (4)	0.1727 (14)	0.0428 (10)	0.131 (3)
H22B	0.3076	1.2125	0.2132	0.051*	0.131 (3)
C23B	0.2982 (8)	1.475 (5)	0.136 (2)	0.0522 (9)	0.131 (3)
H23B	0.3379	1.5149	0.1511	0.063*	0.131 (3)
C24B	0.2575 (17)	1.595 (3)	0.0756 (18)	0.0608 (9)	0.131 (3)
H24B	0.2697	1.7173	0.0506	0.073*	0.131 (3)
C25B	0.1986 (14)	1.536 (4)	0.0526 (14)	0.0573 (9)	0.131 (3)
H25B	0.1711	1.6174	0.0121	0.069*	0.131 (3)
C26B	0.1805 (8)	1.355 (5)	0.090 (2)	0.0561 (9)	0.131 (3)
H26B	0.1408	1.3150	0.0742	0.067*	0.131 (3)
O6B	0.1535 (6)	0.978 (2)	0.1877 (11)	0.077 (5)	0.131 (3)
C9B	0.1998 (8)	1.048 (3)	0.1964 (12)	0.059 (6)*	0.131 (3)

### Atomic displacement parameters (Å^2^)

**Table:**

	*U*^11^	*U*^22^	*U*^33^	*U*^12^	*U*^13^	*U*^23^
O1	0.0475 (9)	0.0382 (10)	0.0499 (10)	0.0048 (8)	0.0003 (8)	0.0000 (8)
O2	0.0472 (9)	0.0429 (10)	0.0581 (10)	−0.0123 (9)	0.0169 (8)	−0.0070 (9)
O3	0.0517 (10)	0.0453 (11)	0.0634 (11)	0.0024 (9)	0.0110 (9)	0.0177 (9)
O4	0.0591 (12)	0.0552 (13)	0.0993 (16)	0.0053 (10)	0.0007 (11)	−0.0290 (12)
O5	0.0679 (12)	0.0612 (13)	0.0925 (15)	−0.0157 (10)	0.0308 (11)	−0.0328 (12)
C1	0.0396 (13)	0.0417 (15)	0.0382 (13)	0.0046 (11)	0.0044 (10)	−0.0013 (12)
C2	0.0507 (15)	0.0372 (14)	0.0445 (13)	−0.0026 (12)	0.0130 (11)	0.0032 (12)
C3	0.0413 (13)	0.0388 (14)	0.0464 (14)	−0.0048 (12)	0.0106 (11)	−0.0036 (12)
C4	0.0388 (13)	0.0474 (15)	0.0432 (14)	0.0005 (12)	0.0064 (10)	−0.0012 (13)
C5	0.0421 (14)	0.0372 (14)	0.0458 (14)	0.0020 (11)	0.0104 (11)	0.0058 (12)
C6	0.0429 (13)	0.0395 (15)	0.0457 (13)	−0.0029 (12)	0.0091 (11)	0.0012 (12)
C7	0.0461 (14)	0.0397 (15)	0.0522 (15)	−0.0017 (13)	0.0141 (12)	−0.0001 (13)
C8	0.0441 (14)	0.0472 (17)	0.0472 (14)	−0.0001 (12)	0.0075 (12)	−0.0017 (13)
C10	0.0406 (13)	0.0410 (15)	0.0482 (14)	0.0024 (12)	0.0128 (11)	0.0026 (12)
C11	0.0518 (16)	0.0458 (17)	0.0748 (19)	0.0068 (13)	0.0128 (14)	0.0017 (15)
C12	0.0577 (18)	0.060 (2)	0.080 (2)	0.0194 (16)	0.0112 (15)	0.0102 (17)
C13	0.0492 (16)	0.082 (2)	0.0601 (17)	0.0045 (17)	0.0057 (14)	0.0026 (17)
C14	0.0582 (17)	0.062 (2)	0.0667 (18)	−0.0016 (16)	0.0022 (14)	−0.0092 (16)
C15	0.0545 (16)	0.0495 (17)	0.0576 (16)	0.0083 (14)	0.0051 (13)	−0.0015 (14)
C16	0.0443 (14)	0.0437 (16)	0.0423 (13)	−0.0037 (12)	0.0065 (11)	0.0015 (12)
C17	0.0471 (15)	0.0463 (17)	0.0584 (16)	−0.0013 (13)	0.0076 (12)	0.0043 (14)
C18	0.0631 (18)	0.0492 (18)	0.0644 (18)	−0.0142 (15)	0.0033 (15)	−0.0014 (15)
C19	0.0600 (18)	0.080 (2)	0.074 (2)	−0.0318 (18)	0.0125 (15)	0.0009 (18)
C20	0.0544 (17)	0.091 (3)	0.085 (2)	−0.0153 (18)	0.0282 (16)	−0.002 (2)
C21	0.0575 (16)	0.0639 (19)	0.0622 (17)	−0.0062 (15)	0.0220 (14)	−0.0067 (15)
C22A	0.052 (3)	0.038 (2)	0.0378 (14)	0.0008 (15)	0.0095 (14)	0.0018 (14)
C23A	0.0564 (19)	0.047 (2)	0.0508 (18)	−0.006 (2)	0.0064 (15)	0.0091 (19)
C24A	0.073 (3)	0.048 (2)	0.059 (2)	−0.010 (2)	0.010 (2)	0.0138 (18)
C25A	0.075 (3)	0.045 (2)	0.0516 (18)	0.0102 (19)	0.015 (2)	0.0108 (16)
C26A	0.0589 (18)	0.058 (3)	0.0516 (19)	0.0195 (19)	0.0123 (15)	0.004 (2)
C27A	0.049 (2)	0.0471 (19)	0.052 (2)	0.0086 (18)	0.0165 (19)	0.0037 (17)
C9A	0.0424 (16)	0.0424 (17)	0.0438 (16)	−0.0015 (15)	0.0103 (13)	0.0017 (14)
O6A	0.0471 (13)	0.0663 (16)	0.0751 (15)	0.0141 (11)	0.0191 (11)	0.0256 (12)
C27B	0.049 (2)	0.0471 (19)	0.052 (2)	0.0086 (18)	0.0165 (19)	0.0037 (17)
C22B	0.052 (3)	0.038 (2)	0.0378 (14)	0.0008 (15)	0.0095 (14)	0.0018 (14)
C23B	0.0564 (19)	0.047 (2)	0.0508 (18)	−0.006 (2)	0.0064 (15)	0.0091 (19)
C24B	0.073 (3)	0.048 (2)	0.059 (2)	−0.010 (2)	0.010 (2)	0.0138 (18)
C25B	0.075 (3)	0.045 (2)	0.0516 (18)	0.0102 (19)	0.015 (2)	0.0108 (16)
C26B	0.0589 (18)	0.058 (3)	0.0516 (19)	0.0195 (19)	0.0123 (15)	0.004 (2)
O6B	0.042 (8)	0.069 (11)	0.119 (13)	−0.003 (8)	0.017 (8)	0.032 (10)

### Geometric parameters (Å, º)

**Table:**

O1—C7	1.357 (3)	C17—H17	0.9300
O1—C1	1.404 (3)	C18—C19	1.3780
O2—C8	1.354 (3)	C18—H18	0.9300
O2—C3	1.407 (3)	C19—C20	1.3780
O3—C9B	1.285 (15)	C19—H19	0.9300
O3—C5	1.394 (3)	C20—C21	1.3780
O3—C9A	1.345 (3)	C20—H20	0.9300
O4—C7	1.194 (3)	C21—H21	0.9300
O5—C8	1.198 (3)	C22A—C23A	1.388 (4)
C1—C6	1.378 (3)	C22A—C27A	1.390 (4)
C1—C2	1.374 (3)	C22A—C9A	1.477 (4)
C2—C3	1.368 (3)	C23A—C24A	1.386 (4)
C2—H2	0.9300	C23A—H23A	0.9300
C3—C4	1.378 (3)	C24A—C25A	1.362 (5)
C4—C5	1.377 (3)	C24A—H24A	0.9300
C4—H4	0.9300	C25A—C26A	1.371 (5)
C5—C6	1.380 (3)	C25A—H25A	0.9300
C6—H6	0.9300	C26A—C27A	1.378 (5)
C7—C10	1.484 (3)	C26A—H26A	0.9300
C8—C16	1.479 (3)	C27A—H27A	0.9300
C10—C11	1.3780	C9A—O6A	1.193 (3)
C10—C15	1.3781	C27B—C22B	1.3780
C11—C12	1.3780	C27B—C26B	1.3781
C11—H11	0.9300	C27B—C9B	1.514 (16)
C12—C13	1.3780	C22B—C23B	1.3780
C12—H12	0.9300	C22B—H22B	0.9300
C13—C14	1.3780	C23B—C24B	1.3780
C13—H13	0.9300	C23B—H23B	0.9300
C14—C15	1.3781	C24B—C25B	1.3780
C14—H14	0.9300	C24B—H24B	0.9300
C15—H15	0.9300	C25B—C26B	1.3780
C16—C17	1.3780	C25B—H25B	0.9300
C16—C21	1.3781	C26B—H26B	0.9300
C17—C18	1.3780	O6B—C9B	1.14 (2)
			
C7—O1—C1	119.26 (19)	C19—C18—H18	120.0
C8—O2—C3	116.22 (19)	C17—C18—H18	120.0
C9B—O3—C5	138.3 (9)	C20—C19—C18	120.0
C9B—O3—C9A	91.9 (8)	C20—C19—H19	120.0
C5—O3—C9A	123.5 (2)	C18—C19—H19	120.0
C6—C1—C2	122.7 (2)	C21—C20—C19	120.0
C6—C1—O1	115.2 (2)	C21—C20—H20	120.0
C2—C1—O1	121.9 (2)	C19—C20—H20	120.0
C1—C2—C3	117.3 (2)	C20—C21—C16	120.0
C1—C2—H2	121.4	C20—C21—H21	120.0
C3—C2—H2	121.4	C16—C21—H21	120.0
C4—C3—C2	122.7 (2)	C23A—C22A—C27A	119.3 (3)
C4—C3—O2	118.6 (2)	C23A—C22A—C9A	116.7 (4)
C2—C3—O2	118.7 (2)	C27A—C22A—C9A	124.0 (4)
C3—C4—C5	117.9 (2)	C22A—C23A—C24A	120.0 (3)
C3—C4—H4	121.1	C22A—C23A—H23A	120.0
C5—C4—H4	121.1	C24A—C23A—H23A	120.0
C4—C5—C6	121.7 (2)	C25A—C24A—C23A	120.0 (3)
C4—C5—O3	116.1 (2)	C25A—C24A—H24A	120.0
C6—C5—O3	122.1 (2)	C23A—C24A—H24A	120.0
C1—C6—C5	117.7 (2)	C24A—C25A—C26A	120.6 (3)
C1—C6—H6	121.2	C24A—C25A—H25A	119.7
C5—C6—H6	121.2	C26A—C25A—H25A	119.7
O4—C7—O1	122.6 (2)	C25A—C26A—C27A	120.3 (3)
O4—C7—C10	125.5 (2)	C25A—C26A—H26A	119.8
O1—C7—C10	111.8 (2)	C27A—C26A—H26A	119.8
O5—C8—O2	122.5 (2)	C22A—C27A—C26A	119.7 (3)
O5—C8—C16	125.2 (2)	C22A—C27A—H27A	120.1
O2—C8—C16	112.3 (2)	C26A—C27A—H27A	120.1
C11—C10—C15	120.0	O6A—C9A—O3	123.3 (3)
C11—C10—C7	117.32 (15)	O6A—C9A—C22A	125.3 (3)
C15—C10—C7	122.53 (15)	O3—C9A—C22A	111.4 (3)
C12—C11—C10	120.0	C22B—C27B—C26B	120.0
C12—C11—H11	120.0	C22B—C27B—C9B	121 (3)
C10—C11—H11	120.0	C26B—C27B—C9B	119 (3)
C11—C12—C13	120.0	C27B—C22B—C23B	120.0
C11—C12—H12	120.0	C27B—C22B—H22B	120.0
C13—C12—H12	120.0	C23B—C22B—H22B	120.0
C12—C13—C14	120.0	C24B—C23B—C22B	120.0
C12—C13—H13	120.0	C24B—C23B—H23B	120.0
C14—C13—H13	120.0	C22B—C23B—H23B	120.0
C15—C14—C13	120.0	C25B—C24B—C23B	120.0
C15—C14—H14	120.0	C25B—C24B—H24B	120.0
C13—C14—H14	120.0	C23B—C24B—H24B	120.0
C14—C15—C10	120.0	C24B—C25B—C26B	120.0
C14—C15—H15	120.0	C24B—C25B—H25B	120.0
C10—C15—H15	120.0	C26B—C25B—H25B	120.0
C17—C16—C21	120.0	C25B—C26B—C27B	120.0
C17—C16—C8	122.56 (15)	C25B—C26B—H26B	120.0
C21—C16—C8	117.44 (15)	C27B—C26B—H26B	120.0
C16—C17—C18	120.0	O6B—C9B—O3	115.8 (15)
C16—C17—H17	120.0	O6B—C9B—C27B	131 (2)
C18—C17—H17	120.0	O3—C9B—C27B	114 (2)
C19—C18—C17	120.0		
			
C6—C1—O1—C7	−125.2 (2)	C4—C5—O3—C9A	−146.3 (2)
C1—O1—C7—O4	−3.8 (4)	C4—C5—O3—C9B	−3.1 (15)
C1—O1—C7—C10	175.05 (18)	C5—O3—C9A—O6A	−0.8 (4)
O1—C7—C10—C11	175.99 (15)	C5—O3—C9A—C22A	178.6 (2)
O1—C7—C10—C15	−8.5 (2)	C5—O3—C9B—O6B	12 (3)
C2—C3—O2—C8	98.2 (3)	C5—O3—C9B—C22B	−169.2 (9)
C3—O2—C8—O5	−0.6 (3)	O3—C9A—C22A—C23A	160.5 (3)
C3—O2—C8—C16	179.54 (18)	O3—C9A—C22A—C27A	−20.7 (4)
O2—C8—C16—C17	14.8 (3)	O3—C9B—C27B—C26B	−172.6 (13)
O2—C8—C16—C21	−164.75 (15)	O3—C9B—C27B—C22B	2 (2)
